# Refractive Error and Visual Functions in Children with Special Needs Compared with the First Grade School Students in Oman

**DOI:** 10.4103/0974-9233.71590

**Published:** 2010

**Authors:** Urmi Vora, Rajiv Khandekar, Sarvanan Natrajan, Khalfan Al-Hadrami

**Affiliations:** Department of Non-Communicable Disease Control, Eye & Ear Health Care, Directorate General of Health Affairs, Ministry of Health (HQ), Muscat, Oman; 1 Department of Public Health, Nizwa, Dhakhiliya Region, Ministry of Health, Oman

**Keywords:** Childhood Blindness, Refractive Error, Rehabilitation, Special Needs Children, Visual Functions

## Abstract

**Background:**

We evaluated the refractive status and visual function of children with special needs (other handicap) in 2010 and compared them with healthy 1^st^ grade school students in Oman.

**Materials and Methods:**

This was a cohort study. Optometrists recorded vision using a logarithm of minimum angle of resolution (LogMAR) chart. Preferential looking method was used for testing 31 children. Cycloplegic refraction was performed on all children. Contrast sensitivity was tested using 2.5%, 10%, and 100% contrast charts. Ocular movement, alignment, and anterior segment were also assessed. A pediatrician reviewed the health records of all the children at the time of their enrollment in this study to determine if the child had been diagnosed with a systemic condition or syndromes. The visual functions were assessed by study investigators. We estimated the rates and the risk of different visual function defects in children with special needs.

**Result:**

The prevalence of refractive error in 70 children (4.7 ± 0.8 years) with special needs (group 1) and 175 normal healthy first grade students (group 2) were 58.5% and 2.9%, respectively. The risk of refractive error was significantly higher in children with special needs [relative risk, 48.1 (95% confidence interval, 17.54–131.8)]. Hyperopia (>1.00 D), myopia (≥ 1.00D) and astigmatism (≥ ±1.00 D) were found in 18.6%, 24.3%, and 27.1%, respectively, in group 1. Six children in this group had defective near vision. Sixteen (80%) children with Down syndrome had refractive error. Seven (50%) children with developmental disorder showed decreased contrast sensitivity.

**Conclusion:**

Prevalence of uncorrected refractive error was much higher in children with special needs. Prevalence of strabismus, nystagmus, and reduced contrast sensitivity was also higher in children with special needs. Early vision screening, visual function assessment, correction of refractive error, and frequent follow-up are recommended.

## INTRODUCTION

The World Health Organization and Vision 2020 included refractive error as a priority in the prevention of childhood blindness and they also recommend low vision care for children.^1^ Accordingly, the assessment of school children for trachoma, anatomic defects, refractive error, and amblyopia at 5–6 years of age and 12–13 years of age, and 15–16 years of age for refractive error has been adopted by many member countries.^2^ Since the yield of vision screening is low and intervention for detected pathologies is often too late, countries that have already adopted screening at school entry should opt for amblyopia screening at an earlier age.^3^ The risk of refractive error among children with special needs has been noted to be higher than that of otherwise healthy children of the same age group.^4^ However, for daily activities, visual functions in addition to the visual acuity should also be addressed. To our knowledge, comparison of the magnitude of refractive error and other visual function defects among children with special needs and healthy children has not been undertaken. Children with other sensory (single or multiple) or mental limitations are defined as children with special needs. In countries, such as Oman where screening of refractive error in schools and visual function assessment of children with special needs are conducted by the health staff, such comparison could be carried out.^5^,^6^

In this study, we compared the status of visual acuity, refractive error, and other visual functions among the children with special needs with that of first grade school children. Based on this, a proposal for improving eye care has been made.

## MATERIALS AND METHODS

We obtained permission from the National Eye Care Committee of Oman to use the health information from the ongoing eye screening of school students for the present study.

This study was conducted between December 2009 and February 2010. Two qualified optometrists, one national supervisor and Arabic speaking teachers were our field investigators.

This was an unmatched type of cohort study. To achieve two-sided confidence interval (CI) of 95% and power of 90% with a ratio of children with special needs (group 1) to the normal healthy children (group 2) as 1:2, we calculated the sample for the study. We used STATCAL of Epi Info 6 (Centers for Disease Control and Prevention, Atlanta, GA, USA) for this purpose. The minimum sample required was 51 children with special needs and 102 healthy children. To compensate for the loss of data, we added another 25% to the sample. Thus the final sample was 64 children with special needs and 127 otherwise healthy children.

The optometrists obtained a detailed history of the children with special needs. This included past and present ocular disorders, medical and surgical treatment, birth weight and term, consanguinity among parents, refractive errors, and ocular disorders in the siblings. This information was confirmed through a health booklet for each student. Visual acuity for distant and near vision was recorded with the help of Lea symbol logarithm of minimum angle of resolution (LogMAR) charts (Good-Lite Co., Elgin, IL, USA) held at 3 m and 40 cm distance from the child, respectively. For children who did not respond to the Lea symbol chart, we recorded visual acuity using preferential looking method and Lea Grating Paddles at 1 m distance.^7^ Those who had visual acuity of <0.3 logMAR for distance or <8 cycles per cm on Lea paddles in the better eye, were considered as having defective vision. The Hirschberg’s test was used to evaluate the fixation pattern.^8^ The cover–uncover test was performed to find and grade strabismus. Ocular movements in all the nine cardinal positions of gaze were noted to rule out strabismus due to muscle restriction or muscle palsy. We noted the type of nystagmus (horizontal, vertical, or oblique). The anterior segment was examined with a flashlight to rule out abnormalities of the eyelid, conjunctiva, cornea, and lens. We also tested pupillary reaction using a penlight.

One drop of homatropine 2% was instilled in each eye. If the cycloplegic effect was not noted in 20 min, another drop was instilled. In group 2, fogging was carried out for all the children and cycloplegia was performed only for those who were identified as having a refractive error and/or accommodation spasm. We used streak retinoscopy (Heine Optotechnik, Herrsching, Germany) for objective refraction at the distance of 66 cm. Refractive error values were compensated for this working distance.

Near vision, contrast visual acuity, and lag of accommodation were tested on subsequent visits after cycloplegic refraction. During these examinations, a trial frame with distance correction in place was used. There was a minimum of 5 days gap between the first and the second visits to prevent the influence of cycloplegia. Contrast visual acuity was recorded with “Lea symbol Low contrast chart” with the 10 M symbol size. This was used for those who responded to the symbol chart during distance vision testing. Hiding Heidi Low contrast Face test was used for those whose visual acuity for distance was recorded on Lea Paddles. Both the tests were performed at 1 m distance. Those who could not identify symbols/grating of 1.25% contrast were recorded as children having defective contrast sensitivity. Lag of accommodation was measured using the Modified Estimation Method (MEM). A streak retinoscope with an attached MEM card was used. Retinoscopy was performed at 40 cm and the child was asked to look at the accommodative pictures on the MEM card and name them. The reflex was neutralized using plus and minus lenses. Generally the lens with which the reflex is neutralized is the net value of the lag of accommodation. Lag of accommodation of +0.25 to +0.50 D is normal. Keeping the age group, systemic condition, and accommodation values in mind, the refractive error corrections (distance and near) were prescribed. Myopia of ≥1.00 D, hyperopia of >1.00 D, and astigmatism of ≥ ±1.00 D were considered for prescription. Information was collected using a standardized data collection form. We used Microsoft Excel^®^ to enter the data. Univariate data analysis using a parametric method was performed with Statistical Package for Social Science (SPSS- 12, SPSS Inc, Chicago, IL, USA). The frequency, prevalence and 95% confidence intervals of refractive error by types were calculated. To compare the results of children with special needs with those of otherwise healthy children, we used odds ratio and the 95% confidence interval. All the children with ocular disorders were given spectacles and low visual aids for free. Teachers and parents of the students who were found to be visually impaired were advised to make the student’s environment low-vision friendly. Children with defective visual functions were advised to undergo periodic eye assessments by pediatric ophthalmologists.

## RESULTS

Our cohort comprised 70 children with special needs in group 1 and 175 healthy children in group 2. The characteristics of these children were compared [Table 1]. The gender difference between the two groups was not significant [odds ratio = 1.55 (95% CI, 0.88–2.75)]. The age differed in both the groups. The children with special needs were younger compared with the normal healthy school children. [difference of mean =1.91 years (95% CI of difference of mean, 1.71–2.11, *P* = 1 × 10^–7^)].

**Table 1:** Demographics and clinical assessment of children with special needs (other disabilities) and healthy 1^st^ grade children in Oman

Variant		Children with special needs		1^st^ grade children		Validation
		N	%	N	%	
Gender	Male	45	64.3	94	53.7	OR = 1.55
	Female	25	35.7	81	46.3	(95% CI, 0.88–2.75)
Clinical diagnosis	ADHD and autism	6	8.6	0	0.0	
	Developmental delay	14	20.0	0	0.0	
	Down syndrome	20	28.6	0	0.0	
	Hearing disorder/speech disability	23	32.9	1	0.6	
	G6PD deficiency	0	0.0	31	17.7	
	Others#	6	8.6	4	2.3	
	Healthy	1	1.4	139	79.4	
Age (years)	Mean	4.73		6.64		Difference of means = 1.91 years 95% CI of diff of mean (1.72–2.1)
	Standard deviation	0.8		0.45		

ADHD: Attention deficit/hyperactivity disorder, G6PD: Glucose-6-phosphate dehydrogenase, CI: Confidence interval, OR: Odds ratio

# Others included

The prevalence of refractive error in children with special needs and the otherwise healthy 1^st^ grade students were 58.5% (95% CI, 47.0–70.0) and 2.9% (95% CI, 0.4–5.4), respectively [Table 2]. The former had a significantly higher risk of refractive error compared with the latter group. [OR = 48.07 (95% CI, 17.5–131.8)]. The rates of hyperopia (>1.00 D), myopia (≥1.00 D), and astigmatism (≥ ±1.00 D) in children with special needs were found to be 18.6%, 24.3%, and 27.1%, respectively, each of which was significantly higher than those for normal children [Table 2].

**Table 2:** Refractive errors in children with special needs (other disabilities) and healthy children in Oman

Refractive error	Special needs (n = 70)		Healthy (n = 175)		OR	95% of OR
	N	%	N	%		
Hypermetropia	24	18.6	2	1.14	39.7	5.08–310
Myopia	17	24.3	4	2.28	19.7	4.32–90.06
Astigmatism	19	27.1	3	1.71	21.4	6.07–75.08
Total	41	58.5	5	2.85	48.07	17.54–131.8

OR: Odds ratio

Of the 20 children with Down syndrome, 16 (80%) had refractive error. Conditions, such as developmental delay, autism, and attention deficit/hyperactivity disorder (ADHD) showed positive association with refractive error. Eleven of the 20 (55.5%) children with these conditions had refractive error. There were 24 children with hearing and speech disorders, 5 (20.8%) of whom had refractive error. Thirty-one healthy school going children had glucose-6-phosphate dehydrogenase (G6PD) deficiency but none had refractive error.

We were able to test the near vision of 34 children with special needs. The rest were in preverbal stage and were tested with other methods. Six (17.6%) of these children had reduced near vision. All the healthy children had normal near vision [Table 3].

**Table 3:** Status of near vision among children with special needs (other disabilities) and healthy children in Oman

Near vision	(M)	Special needs (n = 34*)	Healthy (n = 175)
Normal	5	28	175
Defective	7	1	0
	8	1	0
	9	1	0
	10	2	0
	12	1	0
	Total	6	0

* In 36 children with special needs we could not test near vision as their distance vision could be checked using tests based on preferential looking method

We could not assess the visual function of six children. However, their refractive status and binocular vision were tested. A comparison of contrast sensitivity in the two groups [Table 4] showed that children with special needs had a higher risk of impaired contrast sensitivity than the normal healthy children [OR = 64.3, (95% CI, 8.33–496)]. Of the 14 children with developmental delay, 7 had defective contrast sensitivity.

**Table 4:** Status of contrast sensitivity among children with special needs (other disabilities) and healthy children in Oman

	Special needs (n = 70)		Healthy (n = 175)		OR	95% of OR
	N	%	N	%		
Normal	46		174		64.3	8.33–496
Defective	17	27	1	0.57		
Missing	7		0			

OR: Odds ratio

We found 10 (14.3%) children with special needs showing strabismus compared with only one child among the otherwise healthy school going children. In children with special needs, there were three with nonrefractive esotropia, six with exotropia, and one with dissociated vertical deviation. Three of the children with strabismus had Down syndrome, four had developmental delay, two had acrocephaly, and one had Wilms tumor. There were three (4.3%) special needs children with nystagmus compared with only one child among healthy school going children. The presence of strabismus and nystagmus was greater in special needs children compared with healthy children (odds ratio = 19.6 (95% CI, 4.3–89.5).

Among 22 children with special needs who had refractive error, only three (13.6%) were using spectacles. Five of the 1st grade students were found to have a refractive error, yet, none of these students were using spectacles at the time of this study.

## DISCUSSION

Globally, there are 158 million people with visual impairment due to uncorrected refractive errors. Among 5- to 15-year-old children, uncorrected refractive errors are the main cause of vision impairment.^9^ One of the priorities of the VISION2020 initiative is to manage vision impairment due to uncorrected refractive errors.^1^ Unfortunately, in many developing countries, this issue remains unaddressed.^10^ It is important that in such countries with competing demands, national programs should at least focus on the high-risk groups for visual impairment and disabilities. Children with other disabilities have a significantly higher risk of reduced visual function and refractive error.^4^ The present study provides evidence supporting this earlier observation.

In our study, 58.5% of children with special needs had refractive error. Bankes evaluated 171 mentally handicapped children and reported 52% (95% CI, 44.5–59.5) had refractive error.^11^ Similarly, Gardiner evaluated 60 subjects and found that 45% (95% CI, 32.4–57.6) had refractive error.^12^ Ghising *et al*. reported that 34.4% (95% CI, 26.4 – 42.4) of 134 mentally challenged children had refractive error.^13^ Castañe *et al*. studied the type of refractive error and found that among 46 mentally challenged children, 58.7% had hyperopia (58.7%), 21.7% had myopia (21.7%), and 19.5% had astigmatism, indicating the significantly high proportion of refractive error in these subjects.^14^ All the aforementioned studies were carried out on mentally challenged children of 5–16 years of age. In contrast, our study had children with different types of clinical conditions. One third of the population in our study had hearing and speech disorder and only five (21.7%) of them had refractive error. Gogate *et al*. conducted a study in India and found an 18.5% prevalence of refractive error among children with hearing impairment.^15^ This difference in the rates of refractive error could be attributed to the different groups in the two studies, that is, one Indian population and the other Arab population of children.

We found that 80% of children with Down syndrome in our study had refractive error. Kim *et al*. found that 86.5% of 172 children with Down syndrome had refractive errors.^16^ In another study by Stephen *et al*., 43% of 81 children with Down syndrome had a refractive error.^17^ Refractive error and strabismus were noted in 12% and 19%, respectively, among 57 children with Down syndrome in Turkey.^18^ In our cohort, 80% of the 20 children with Down syndrome had a refractive error. Due to the small sample, the high rates of refractive error that were found in our study should be interpreted with caution. Further studies for ocular manifestation in children with Down syndrome in Oman are recommended.

It is interesting to note that among 31 children of the 175 otherwise healthy children in the 1st grade, there was a G6PD blood disorder and none of them had refractive error. In contrast, among 144 children without G6PD, five had refractive error. This observation needs further confirmation.

In our study, 14.3% of children with special needs had strabismus. Only three children with Down syndrome had strabismus. Other studies had estimated the incidence of strabismus in children with Down syndrome ranging from 19% to 30%.^18^,^19^ Strabismus could be the result of amblyopia or amblyogenic factor but binocularity is usually lost. Binocular vision disruption at earlier age affects the normal development of the child. It also hinders the learning process.^20^ Therefore, strabismus should be corrected by pediatric ophthalmologists and appropriate orthoptic management should be attempted. Children with nystagmus may have reduced vision and they may also have difficulties in fixating on objects. This could also be an obstacle in the learning process. In our study, we found 4.3% of children with nystagmus.

Contrast sensitivity is one of the important visual functions for daily living. Contrast sensitivity defects were noted in children with special needs in our study. Sandfeld *et al*.^21^ and Nielson *et al*.^22^ found that children with developmental delay had decreased contrast sensitivity. In our study, 7 of 14 children with developmental delay had reduced contrast sensitivity. The association of delayed development with the prevalence of refractive error and defective contrast sensitivity should be studied further with a larger sample.

Six children with special needs were absent for further visual function assessment. We assumed that their visual function was normal. If they showed visual function abnormality, the rate of visual function defects in children with special needs would be an underestimate, and thus the comparison of the two groups would have shown a wider gap.

Akinci *et al*. noted that as many as 97.4% of children with Down syndrome had ocular findings.^23^ This was in contrast to 42.4% of persons without Down syndrome with ocular findings.^23^ In an earlier study by Maino *et al*., the incidence of ocular defects was high among mentally challenged children and the importance of their eye care was highlighted.^24^ Similarly, in our study the incidence of refractive error in children with special needs was higher compared with the healthy school going children. Children with special needs have other disabilities that are prioritized, and the need for eye care often remains unnoticed or neglected. Refractive error if left untreated, results in visual disability adding to compromised quality of life, yet is avoidable. Refractive error correction at early age prevents amblyopia and thus prevents the child from spending the productive years of life with avoidable blindness. Eye health care providers should educate parents and teachers about the need for early eye screening.

Vision screening programs in many countries are undertaken for early detection and care of eye problems in children. Usually such eye screening is performed by the health care providers and further management of children with defective vision is carried out by optometrists at vision centers. This strategy might not work for the children with special needs. We suggest that optometrists/low vision care experts should perform ocular examination in the day care centers or homes of children with special needs as they are more cooperative in a familiar setting. As teachers and caregivers are known to the children with special needs, they can help the low vision therapist to achieve the child’s cooperation. This was possible for the authors because they had a portable Visual function assessment kit (Good-Lite Co, Elgin, IL, USA).

In a resource-intensive country, such as Oman, 13.6% of children with special needs with refractive errors were using spectacles, which is less than desired. In India the use of spectacles among hearing impaired children with refractive error was as low as 6%.^15^ This low uptake could be due to inadequate counseling by optometrists or noncompliance in using spectacles. Periodic screening of children with special needs and proper counseling could address this problem.

Children with special needs in our cohort were 1.9 years younger than the otherwise healthy 1st grade students. Routinely, vision screening of healthy children is conducted at the time of their enrollment (previous academic year). If we take this time of assessment as an index for calculating the age of child for vision testing in the 1st grade, the age difference in two groups was not significant. It should be noted that a detailed visual function assessment and refractive error management as part of our study was done in the later part of the same academic year.

Among hearing impaired children, ocular defects, if present, markedly compromise the quality of life due to the burden of double disabilities in this vulnerable group.^25^ A review of survey data in Oman showed that combination of visual and hearing impairment was significantly high.^26^ A visual function assessment of children with severe and profound hearing impairment found that the risk for visual defect was 64%.^26^ A study in India also noted that 24% children with hearing impairment had ocular defects.^15^

Prevalence of uncorrected refractive error, especially astigmatism was much higher in children with special needs compared with the 1^st^ grade children who were otherwise healthy. Prevalence of strabismus, nystagmus, and defective contrast sensitivity was also higher in children with special needs. Children with special needs are at a much higher risk of eye and vision problems than normal healthy children, and thus children with all forms of special needs not only have equal rights to optometric services but have a greater need than the rest of the population. These children require more attention of the National Eye Health care program.
